# Dentate total molecular layer interneurons mediate cannabinoid-sensitive inhibition

**DOI:** 10.1002/hipo.22419

**Published:** 2015-02-09

**Authors:** Jiandong Yu, Bogumila Swietek, Archana Proddutur, Vijayalakshmi Santhakumar

**Affiliations:** 1 Department of Neurology and Neurosciences, Rutgers New Jersey Medical SchoolNewark, New Jersey; 2 Department of Pharmacology and Physiology, Rutgers New Jersey Medical SchoolNewark, New Jersey

**Keywords:** dentate gyrus, interneuron, inhibition, cannabinoid

## Abstract

Activity of the dentate gyrus, which gates information flow to the hippocampus, is under tight inhibitory regulation by interneurons with distinctive axonal projections, intrinsic and synaptic characteristics and neurochemical identities. Total molecular layer cells (TML-Cs), a class of morphologically distinct GABAergic neurons with axonal projections across the molecular layer, are among the most frequent interneuronal type in the dentate subgranular region. However, little is known about their synaptic and neurochemical properties. We demonstrate that synapses from morphologically identified TML-Cs to dentate interneurons are characterized by low release probability, facilitating short-term dynamics and asynchronous release. TML-Cs consistently show somatic and axonal labeling for the cannabinoid receptor type 1 (CB_1_R) yet fail to express cholecystokinin (CCK) indicating their distinctive neurochemical identity. In paired recordings, the release probability at synapses between TML-Cs was increased by the CB_1_R antagonist AM251, demonstrating baseline endocannabinoid regulation of TML-C synapses. Apart from defining the synaptic and neurochemical features of TML-Cs, our findings reveal the morphological identity of a class of dentate CB_1_R-positive neurons that do not express CCK. Our findings indicate that TML-Cs can mediate cannabinoid sensitive feed-forward and feedback inhibition of dentate perforant path inputs. © 2015 The Authors Hippocampus Published by Wiley Periodicals, Inc.

The dentate gyrus is known for its laminar inputs with commissural and associational fibers targeting the inner molecular layer (IML) while medial and lateral perforant path project to the middle and outer molecular layers (Frotscher, 1991). Inhibitory projections share a similar laminar structure with parvalbumin-expressing fast-spiking basket cell (FS-BC) axons in the granule cell layer, hilar commissural-associational pathway-associated (HICAP) cells and CCK-expressing neurons projecting to the IML and somatostatin-expressing hilar-perforant pathway-associated (HIPP) cells terminating in the outer molecular layer (Buckmaster et al., 2002; Hefft and Jonas, 2005). Additionally, there exists a class of total molecular layer cells (TML-Cs) with axonal projection across the entire dentate molecular layer (Soriano and Frotscher, 1993; Buckmaster and Schwartzkroin, 1995; Mott et al., 1997). TML-Cs have high axonal density in the middle molecular layer and are positioned to mediate feedback inhibition of medial perforant path inputs (Soriano and Frotscher, 1993; Hosp et al., 2014). Although described as relatively numerous (Mott et al., 1997; Zhang and Buckmaster, 2009), little is known about TML-Cs aside from morphology and basic firing characteristics (Soriano and Frotscher, 1993; Mott et al., 1997; Hosp et al., 2014). Determining the neurochemical identity and synaptic characteristics of TML-Cs is essential to defining their role in the dentate circuit.

A related intriguing issue concerns the expression of cannabinoid receptor type 1 (CB_1_R) in the dentate molecular layer. CB_1_R is present in certain glutamatergic and GABAergic terminals in the dentate IML (Monory et al., 2006; Morozov et al., 2009). Among glutamatergic terminals, CB_1_R is present exclusively in mossy cell axons and excluded from perforant path inputs (Monory et al., 2006). Although axons of CCK-positive neurons known to express CB_1_R are localized to the IML, deletion of CB_1_R from glutamatergic terminals reveals CB_1_R labeled axons distributed across the entire molecular layer (Monory et al., 2006). The axonal distribution of TML-Cs raises the possibility that these neurons may have CB_1_R-positive axons and undergo cannabinoid-modulation of synaptic release. This study was conducted to identify the synaptic features and CB_1_R expression profile of dentate TML-Cs in order to aid further analysis of their contribution to circuit function.

Briefly, horizontal brain slices (300 μM) were prepared from male, Wistar rats >30 days old under protocols approved by Rutgers-NJMS, Newark, NJ, IACUC. Neurons in the subgranular hilus were patched using microelectrodes containing equal concentrations of KCl and K-gluconate and 0.2% biocytin (Proddutur et al., 2013; Yu et al., 2013). Intrinsic properties were determined from responses to 1.5 sec current injections from a -70 mV holding potential. Only neurons with dendrites in the hilus and molecular layer and total molecular layer axons on post-hoc morphological analysis were included as TML-Cs (Soriano and Frotscher, 1993). Neurons with non-adapting high frequency firing with axons in the granule cell layer and co-labeling for parvalbumin were considered FS-BCs (Yu et al., 2013). Neurons with axons predominantly in the IML and adapting firing pattern were considered HICAP cells. For paired recordings, presynaptic interneurons were stimulated with 2−8 current pulses (3 ms, 700-1100pA) at 50 Hz every 10 s in current-clamp mode while postsynaptic neurons were voltage-clamped at −70 mV. Synaptic events were analyzed using Clampfit (Molecular Devices). Following recordings, slices were fixed 4% paraformaldehyde and immunolabeled with anti-CCK (1 : 1,000, monoclonal mouse, courtesy of G. Ohning, CURE, UCLA), anti-CB_1_R (1 : 1,000, polyclonal guinea pig, Frontier Science) or anti-PV antibody (1.5 : 1,000, polyclonal rabbit, Swant) using previously described protocols (Yu et al., 2013). Some experiments included resectioned or perfusion-fixed sections (50 μm). Biocytin staining was revealed using Alexa 594-conjugated streptavidin. Images were obtained using a Nikon A1R laser confocal microscope (1.2 NA 60X water objective) and used for morphological reconstruction (Neurolucida). Data are presented as mean ± s.e.m. *P* < 0.05 by *t*-test or Mann-Whitney U test (for data that failed normality test) were considered significant.

TML-Cs were identified by the relatively sparse axonal distribution in the dentate molecular layer with occasional hilar collaterals (Figs. 1A and 2A), as shown before (Soriano and Frotscher, 1993; Buckmaster and Schwartzkroin, 1995; Mott et al., 1997). Consistent with previous reports (Soriano and Frotscher, 1993), TML-Cs had relatively small somata (247.8 ± 27.2 μm^2^, *n* = 12 cells) compared to FS-BCs (350.8 ± 19.3 μm^2^, *n* = 12 cells, *P* < 0.05) and aspiny dendrites extending into both the molecular layer and hilus. Molecular layer dendrites of TML-Cs extended to the hippocampal fissure (Fig. 1A). Intrinsic physiology of TML-Cs was characterized by accommodating firing pattern, distinct from the non-adapting firing of FS-BCs (Fig. 1B). TML-Cs had lower maximum discharge frequency (Fig. 1C, frequency in Hz at 800 pA current injection 36.2 ± 5.5, *n* = 14 cells) and higher input resistance (*R*_in_) (Figs. 1B,D, *R*_in_ in MΩ, 222.6 ± 17.1, *n* = 14 cells) than FS-BCs (frequency in Hz: 112.1 ± 7.5, *n* = 12 R_in_ in MΩ, 93.0 ± 10.1, *n* = 12 cells, reported in Proddutur et al., 2013; Yu et al., 2013). Additionally, unlike FS-BCs, TML-Cs showed spike frequency adaptation (Figs. 1B,E, Ratio ISI_first/last_, TML-C: 0.33 ± 0.04, *n* = 14 cells, FS-BC: 0.84 ± 0.04, *n* = 12 cells) during sustained depolarization and a greater membrane potential sag during hyperpolarization (Sag ratio, TML-C: 0.86 ± 0.02, *n* = 14 cells, FS-BC: 0.96 ± 0.01, *n* = 12 cells, *P* < 0.05 by *t*-test). Although TML-C intrinsic properties such as adapting firing pattern, high input resistance and presence of membrane sag resemble those of CCK expressing HICAP cells (Mott et al., 1997; Savanthrapadian et al., 2014), they were morphologically distinguished from HICAP cells based on axonal distribution in the middle and outer molecular layers.

**Figure 1:**
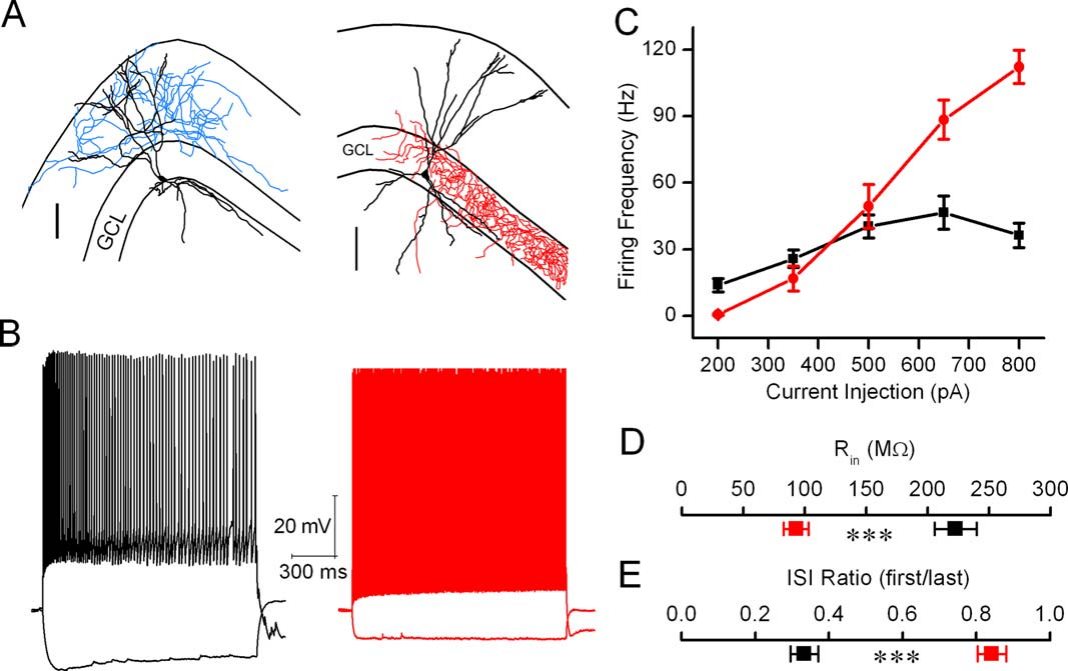
Morphological and physiological identification of TML cells. A: Neurolucida reconstruction of a TML cell (left panel) shows axon collaterals (blue) in all three molecular layers and dendrites (black) extending to the fissure. Scale bar, 100 μm. Reconstruction of an FS-BC (right panel) with axon (red) in granule cell layer (GCL). B: Membrane voltage traces show adapting firing pattern in a TML-C (left panel) and fast-spiking, non-adapting firing in a FS-BC (right panel, in red) during a +500 pA current injection. Note the larger membrane hyperpolarization and presence of membrane sag in the TML-C during −100 pA current injections in TML-C and FS-BCs. C: Summary of current-firing characteristics of TML-Cs and FS-BCs. D,E: Summary plots compare TML-C and FS-BC input resistance measured during a −100 pA current injection (D) and adaptation ratio (E) measured as the ratio between first and last inter spike intervals in response to a 500 pA current injection for 1,500 ms.

**Figure 2:**
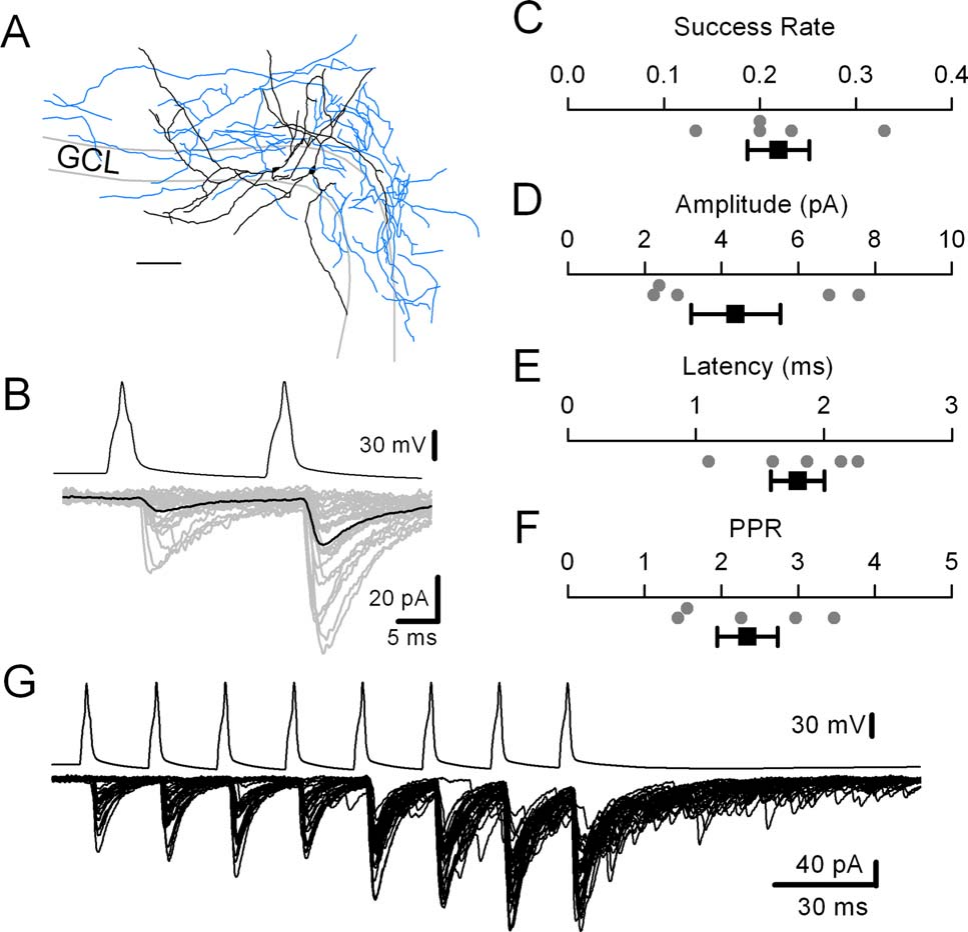
Characteristics of TML-C synaptic connections. A: Neurolucida reconstruction of a pair of TML-Cs shows axon collaterals (blue) in all three molecular layers. Scale bar, 100 μm. B: Voltage traces (top) illustrates action potentials in presynaptic TML-C (left panel, 2 APs at 50 Hz). Overlay of individual responses evoked by 30 consecutive sweeps (in gray) and the average current response (black) in the postsynaptic TML-C are shown. Note the short term facilitation. C−F: Summary plots of TML-C synaptic success rate (C), amplitude (D), latency (E) and paired pulse ratio (F). (G) Voltage trace (top trace) shows action potentials (8 Aps at 50 Hz) in a presynaptic TML-C. Individual current responses evoked by 30 consecutive sweeps (bottom trace) in the postsynaptic TML-C are illustrated. Note the paired and multi-pulse facilitation and asynchronous synaptic release, particularly after the end of presynaptic firing. Both neurons in the paired recordings in B and G were morphologically identified as TML-Cs.

In paired interneuronal recordings, TML-C activation evoked monosynaptic responses in hilar interneurons including TML-Cs (Figs. 2A,B, *n* = 5 pairs) and FS-BCs (*n* = 7 pairs, not shown). Compared to synapses between FS-BCs (7 pairs), unitary inhibitory postsynaptic currents (uIPSCs) between TML-Cs (5 pairs) were characterized by low release probability (Figs. 2B,C; success rate, TML-C: 22 ± 3%; FS-BC: 86 ± 6%, *P* < 0.05) and amplitude (Figs. 2B,D; uIPSC amplitude including failures in pA, TML-C: 4.4 ± 1.2; FS-BC: 185 ± 98.8, *P* < 0.05 by U test). TML-C uIPSC amplitude potency excluding failures was 19.5 ± 3.9 pA. Similarly, 20–80% rise time (in ms, TML-C: 0.96 ± 0.19; FS-BC: 0.39 ± 0.05, *P* < 0.05 by U test) and decay times (*τ*_decay_ in ms, TML-C: 8.28 ± 2.08; FS-BC: 3.23 ± 0.61, *P* < 0.05 by U test) were slower than in FS-BC synapses. TML-C synapses had longer latency (Fig. 2E; in ms, TML-C: 1.8 ± 0.2, FS-BC: 0.8 ± 0.1, *P* < 0.05 by U test) and higher CV of latency (TML-C: 0.41 ± 0.14, FS-BC: 0.14 ± 0.02, *P* < 0.05 by U test) than FS-BCs, which is similar to data from IPSCs between presumed HICAP cells (Savanthrapadian et al., 2014). Moreover, TML-C synapses showed paired and multi-pulse facilitation (Figs. 2B,G) rather than the depression between FS-BCs (Savanthrapadian et al., 2014). TML-Cs exhibit both synchronous and asynchronous release in response to activation at 50 Hz (Fig. 2G), which is similar to the asynchronous release reported in HICAP cells and in CCK-expressing neurons in the hippocampus (Ali and Todorova, 2010; Savanthrapadian et al., 2014; Szabo et al., 2014). Synaptic responses from TML-Cs were blocked by 10 μM SR95531 (*n* = 3 pairs, not shown) indicating that inhibition was mediated by GABA_A_ receptors. These data demonstrate that, like intrinsic properties, synaptic characteristics of morphologically identified TML-Cs are distinct from FS-BC and similar to dentate HICAP cells (Savanthrapadian et al., 2014). To our knowledge, these data represent the first functional characterization of TML-C synapses.

Since TML-Cs show asynchronous synaptic release characteristic of CB_1_R-expressing neurons, we examined if TML-Cs express CB_1_R. Immunostaining for CB_1_R and CCK in dentate sections revealed distinct CB_1_R positive fibers in the middle and outer molecular layers that did not co-localize with CCK (not shown), in addition to IML axonal fibers co-labeled for CB_1_R and CCK. Since perforant path inputs do not exhibit cannabinoid modulation (Chancey et al., 2014) and CB_1_R-positive mossy cell axons are restricted to the IML (Monory et al., 2006), our immunostaining data suggest that interneurons with axons spanning the molecular layer likely express CB_1_R. Direct examination of TML-Cs filled with biocytin during recordings revealed somatic labeling for CB_1_R (Figs. 3A–D, reconstruction of the cell in Figure 1A confirms TML-C morphology, *n* = 11 of 11 cells tested). However, TML-Cs lacked somatic or dendritic labeling for CCK (Figs. 3A–D, *n* = 8) despite prominent CCK immunoreactivity in adjacent neurons (Fig. 3C, inset panels to right of 3D show colocalization of CB_1_R in the CCK expressing cell). Since TML cells showed somatic labeling for CB_1_R, and neurons with IML axons recorded under similar conditions were co-labeled for CCK and CB_1_R (data not shown), it is unlikely that the recording conditions resulted in absence of CCK expression in TML-Cs. Thus, TML-Cs are morphologically distinct from HICAP cells and can be neurochemically distinguished from CCK- and CB_1_R-positive neurons with IML axons. In addition to the soma, molecular layer axon collaterals of TML-Cs were labeled with CB_1_R (Figs. 3E–J, panels E-G are from cell in 3A, H, I, and J are from two different cells) indicating that TML-Cs may contribute to the expansive CB_1_R labeling in dentate GABAergic terminals (Monory et al., 2006; Magloczky et al., 2010).

**Figure 3:**
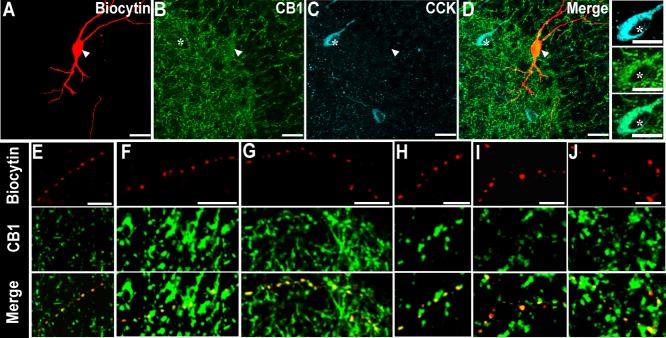
CB_1_R expression in TML-C somata and axon collaterals. A−D: Confocal images at 60× of the TML cell illustrated in Figure 1A shows the biocytin filled soma (A), labeling for CB_1_R (B), CCK (C), and merge (D). Arrowhead denotes biocytin labeled cell co-labeled for CB_1_R. * marks CCK expressing neuron co-labeled for CB_1_R. Note the labeling for CB_1_R and not CCK in the biocytin labeled soma and presence of CCK labeling in adjacent cells in C. Insets to right of D shows colocalization of CB_1_R in a CCK positive cell (*) adjacent to the recorded cell. Scale bar, 25 μm. GC: granule cell layer. E−J: Confocal images of biocytin-filled axons (red, top panel) from the same cell as in A (E−G) and two additional TML cells (H−J) show CB_1_R labeling (middle panel) in the merged image (bottom panel). Scale bar, 10 μm.

Activation of CB_1_R, in the presynaptic terminals of CCK-positive interneurons, leads to a reduction in probability of synaptic release from CCK neurons to principal cells and interneurons (Freund, 2003; Armstrong and Soltesz, 2012). The characteristic baseline and activity-dependent cannabinoid modulation of synaptic release has been used to distinguish between inhibition from CCK-positive interneurons and FS-BCs that lack cannabinoid modulation. Given the expression of CB_1_R on TML-C axons we examined whether TML-C synapses show CB_1_R-dependent modulation synaptic release. In 4 out of 4 TML-C pairs tested, the CB_1_R antagonist, AM251 (10 μM) consistently and reversibly enhanced the synaptic success rate (Figs. 4A,B, 194.2 ± 12.0% of baseline, 4 pairs) and uIPSC amplitude (Figs. 4A,B, 195.0 ± 29.8% of baseline, 4 pairs). In two TML-C pairs tested, the CB_1_R agonist WIN-55212 (10 μM) reduced the probability of synaptic release (from 0.23 to 0.13, and from 0.2 to 0.1 respectively) suggesting that CB_1_R-modulation of TML-C synaptic release is bidirectional. Additionally, release probability at TML-C synapses on FS-BCs was also enhanced by AM251 (4 pairs, not shown). Together these data demonstrate functional, baseline endocannabinoid modulation of unitary TML-C synapses.

**Figure 4:**
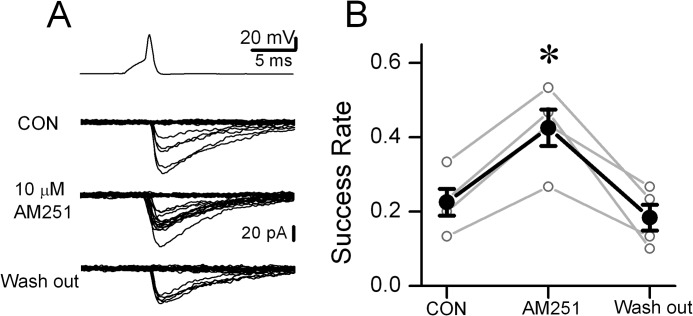
CB_1_R antagonist modulates release at synapses between TML cells. Consecutive unitary IPSCs traces evoked by presynaptic firing at synapses between TML-Cs in control aCSF (baseline), during perfusion of the CB1 antagonist AM251 (10 μM) and during drug washout. Note that AM251 reduced the number of failures. B: Summary data show the effect of AM251 on the success rate of TML-C synapses and recovery after drug wash out (individual pairs are in gray and average is in black, *P < 0.05, paired *t*-test, compared to baseline).

This study constitutes the first detailed physiological and neurochemical characterization of dentate TML cells, a class of GABAergic neurons in the hilar-granule cell layer border. Although TML-Cs were initially described over 20 years ago (Soriano and Frotscher, 1993) and have been observed during dentate interneuronal recordings (Mott et al., 1997; Zhang and Buckmaster, 2009; Hosp et al., 2014) their synaptic properties have not been examined. Apart from synaptic contacts on presumed granule cells (Soriano and Frotscher, 1993), we find that TML-Cs innervate other interneurons including TML-Cs and FS-BCs. Functionally, TML-C synapses show high temporal jitter in latency and asynchronous release suggesting that TML-Cs, like CCK-positive cells, contribute to prolonged synaptic inhibition during sustained network activity rather than the temporally precise inhibition of FS-BCs (Freund, 2003; Armstrong and Soltesz, 2012; Savanthrapadian et al., 2014). Moreover, the low reliability and multi-pulse facilitation also favor a more sustained role for TML-Cs in dentate inhibition. While we directly demonstrate CB_1_R-sensitive synapses between TML-Cs, release at TML-C synapses on granule cells and other interneurons are also likely modulated by CB_1_Rs. Thus, in addition to the commissural-associational inputs in the IML that are regulated by asynchronous inhibition from HICAP cells, perforant path inputs in the middle and outer molecular layers are potentially regulated by feed-forward and feedback inhibition from TML-Cs with facilitating synaptic dynamics.

A salient finding of this study is that TML-Cs, show somatic and axonal expression of CB_1_R. TML-C synapses exhibit low release probability that was enhanced by the CB_1_R inverse agonist AM251 (Fig. 4B) indicating baseline CB_1_R-dependent suppression of release as reported in cortical and hippocampal CB_1_R-expressing neurons (Losonczy et al., 2004; Foldy et al., 2006). The presence of CB_1_R and not CCK in TML-Cs is distinct from both HICAP cells expressing CCK designated as CCK-positive basket cells (Hefft and Jonas, 2005; Savanthrapadian et al., 2014) and HICAP cells lacking CB_1_R-modulation (Liu et al., 2014). Moreover, unlike hippocampal neurons that co-localize CCK and CB_1_R (Ali and Todorova, 2010; Lee et al., 2010; Szabo et al., 2014), TML-Cs uniquely express CB_1_R and lack CCK. While earlier studies have suggested the presence of CB_1_R in dentate interneurons lacking CCK (Hajos et al., 2000; Morozov et al., 2009), our study provides the first morphological and functional identification of these neurons. By demonstrating that neurons with dentate molecular layer axons express CB_1_R and not CCK, our findings shed light on differential distribution of CCK- and CB_1_R-expressing inhibitory axonal collaterals across the dentate molecular layer (Hefft and Jonas, 2005; Magloczky et al., 2010; Monory et al., 2006). More importantly, our data demonstrate that CB_1_R-sensitive inhibition in the dentate cannot be considered synonymous with inhibition from CCK-expressing interneurons.

## References

[b1] Ali AB, Todorova M (2010). Asynchronous release of GABA via tonic cannabinoid receptor activation at identified interneuron synapses in rat CA1. Eur J Neurosci.

[b2] Armstrong C, Soltesz I (2012). Basket cell dichotomy in microcircuit function. J Physiol.

[b3] Buckmaster PS, Schwartzkroin PA (1995). Interneurons and inhibition in the dentate gyrus of the rat in vivo. J. Neurosci.

[b4] Buckmaster PS, Yamawaki R, Zhang GF (2002). Axon arbors and synaptic connections of a vulnerable population of interneurons in the dentate gyrus in vivo. J Comparative Neurol.

[b5] Chancey JH, Poulsen DJ, Wadiche JI, Overstreet-Wadiche L (2014). Hilar mossy cells provide the first glutamatergic synapses to adult-born dentate granule cells. J Neurosci.

[b6] Foldy C, Neu A, Jones MV, Soltesz I (2006). Presynaptic, activity-dependent modulation of cannabinoid type 1 receptor-mediated inhibition of GABA release. J. Neurosci.

[b7] Freund TF (2003). Interneuron diversity series: Rhythm and mood in perisomatic inhibition. Trends Neurosci.

[b8] Frotscher M (1991). Target cell specificity of synaptic connections in the hippocampus. Hippocampus.

[b9] Hajos N, Katona I, Naiem SS, MacKie K, Ledent C, Mody I, Freund TF (2000). Cannabinoids inhibit hippocampal GABAergic transmission and network oscillations. Eur J Neurosci.

[b10] Hefft S, Jonas P (2005). Asynchronous GABA release generates long-lasting inhibition at a hippocampal interneuron-principal neuron synapse. Nat Neurosci.

[b11] Hosp JA, Struber M, Yanagawa Y, Obata K, Vida I, Jonas P, Bartos M (2014). Morpho-physiological criteria divide dentate gyrus interneurons into classes. Hippocampus.

[b12] Lee SH, Foldy C, Soltesz I (2010). Distinct endocannabinoid control of GABA release at perisomatic and dendritic synapses in the hippocampus. J Neurosci.

[b13] Liu YC, Cheng JK, Lien CC (2014). Rapid dynamic changes of dendritic inhibition in the dentate gyrus by presynaptic activity patterns. J Neurosci.

[b14] Losonczy A, Biro AA, Nusser Z (2004). Persistently active cannabinoid receptors mute a subpopulation of hippocampal interneurons. Proc Natl Acad Sci USA.

[b15] Maglóczky Z, Tóth K, Karlócai R, Nagy S, Eross L, Czirják S, Vajda J, Rásonyi G, Kelemen A, Juhos V, P Halász, K Mackie, TF Freund (2010). Dynamic changes of CB1-receptor expression in hippocampi of epileptic mice and humans. Epilepsia.

[b16] Monory K, Massa F, Egertová M, Eder M, Blaudzun H, Westenbroek R, Kelsch W, Jacob W, Marsch R, Ekker M, J Long, JL Rubenstein, S Goebbels, KA Nave, M During, M Klugmann, B Wölfel, HU Dodt, W Zieglgänsberger, CT Wotjak, K Mackie, MR Elphick, G Marsicano, B Lutz (2006). The endocannabinoid system controls key epileptogenic circuits in the hippocampus. Neuron.

[b17] Morozov YM, Torii M, Rakic P (2009). Origin, early commitment, migratory routes, and destination of cannabinoid type 1 receptor-containing interneurons. Cereb Cortex.

[b18] Mott DD, Turner DA, Okazaki MM, Lewis DV (1997). Interneurons of the dentate-hilus border of the rat dentate gyrus: Morphological and electrophysiological heterogeneity. J Neurosci.

[b19] Proddutur A, Yu J, Elgammal FS, Santhakumar V (2013). Seizure-induced alterations in fast-spiking basket cell GABA currents modulate frequency and coherence of gamma oscillation in network simulations. Chaos.

[b20] Savanthrapadian S, Meyer T, Elgueta C, Booker SA, Vida I, Bartos M (2014). Synaptic properties of SOM- and CCK-expressing cells in dentate gyrus interneuron networks. J Neurosci.

[b21] Soriano E, Frotscher M (1993). GABAergic innervation of the rat fascia dentata: A novel type of interneuron in the granule cell layer with extensive axonal arborization in the molecular layer. J Comp Neurol.

[b22] Szabó GG, Papp OI, Máté Z, Szabó G, Hájos N (2014). Anatomically heterogeneous populations of CB1 cannabinoid receptor-expressing interneurons in the CA3 region of the hippocampus show homogeneous input-output characteristics. Hippocampus.

[b23] Yu J, Proddutur A, Elgammal FS, Ito T, Santhakumar V (2013). Status epilepticus enhances tonic GABA currents and depolarizes GABA reversal potential in dentate fast-spiking basket cells. J Neurophysiol.

[b24] Zhang W, Buckmaster PS (2009). Dysfunction of the dentate basket cell circuit in a rat model of temporal lobe epilepsy. J Neurosci.

